# Spatiotemporal expression of HMGB2 regulates cell proliferation and hepatocyte size during liver regeneration

**DOI:** 10.1038/s41598-022-16258-4

**Published:** 2022-07-13

**Authors:** Koichi Yano, Narantsog Choijookhuu, Makoto Ikenoue, Fidya, Tomohiro Fukaya, Katsuaki Sato, Deokcheol Lee, Noboru Taniguchi, Etsuo Chosa, Atsushi Nanashima, Yoshitaka Hishikawa

**Affiliations:** 1 Department of Anatomy, Histochemistry and Cell Biology, Faculty of Medicine, University of Miyazaki, 5200 Kihara, Kiyotake, Miyazaki, 889-1692 Japan; 2 Department of Surgery, Faculty of Medicine, University of Miyazaki, Miyazaki, 889-1692 Japan; 3 Division of Immunology, Department of Infectious Diseases, Faculty of Medicine, University of Miyazaki, Miyazaki, Japan; 4 Department of Orthopaedic Surgery, Faculty of Medicine, University of Miyazaki, 5200 Kihara, Kiyotake, Miyazaki, 889‑1692 Japan; 5 Department of Orthopaedic Surgery, Graduate School of Medical and Dental Sciences, Kagoshima University, 8‑35‑1 Sakuragaoka, Kagoshima, 890‑8520 Japan

**Keywords:** Hepatocytes, Cell growth

## Abstract

Liver regeneration is an extraordinarily complex process involving a variety of factors; however, the role of chromatin protein in hepatocyte proliferation is largely unknown. In this study, we investigated the functional role of high-mobility group box 2 (HMGB2), a chromatin protein in liver regeneration using wild-type and HMGB2-knockout (KO) mice. Liver tissues were sampled after 70% partial hepatectomy (PHx), and analyzed by immunohistochemistry, western blotting and flow cytometry using various markers of cell proliferation. In WT mice, hepatocyte proliferation was strongly correlated with the spatiotemporal expression of HMGB2; however, cell proliferation was significantly delayed in hepatocytes of HMGB2-KO mice. Quantitative PCR demonstrated that *cyclin D1* and *cyclin B1* mRNAs were significantly decreased in HMGB2-KO mice livers. Interestingly, hepatocyte size was significantly larger in HMGB2-KO mice at 36–72 h after PHx, and these results suggest that hepatocyte hypertrophy appeared in parallel with delayed cell proliferation. In vitro experiments demonstrated that cell proliferation was significantly decreased in HMGB2-KO cells. A significant delay in cell proliferation was also found in HMGB2-siRNA transfected cells. In summary, spatiotemporal expression of HMGB2 is important for regulation of hepatocyte proliferation and cell size during liver regeneration.

## Introduction

The liver is a unique organ with a high regenerative capacity. Understanding the molecular mechanisms of liver regeneration is clinically important because partial hepatectomy (PHx) and liver transplantation are curative treatments for hepatocellular carcinoma and severe liver diseases^1,2^. After PHx, the liver mass returns to preoperative size within 3 months in humans, a process that occurs in 7–10 days in rodents^3^. The remnant liver exhibits different responses depending on the amount of resected liver. In rodents, both cell proliferation and hypertrophy appear after 70% PHx, whereas only compensatory hypertrophy is observed after 30% PHx^4^. Therefore, 70% PHx is the ideal model to investigate the role of cell number and size in liver regeneration.

Although liver tissue consists of several cell types, hepatocytes are the major cell type, representing 80% of the liver by weight and 70% by cell number^5^. In normal liver, hepatocytes remain in a quiescent state; in contrast, hepatocytes exhibit a massive, synchronized cell proliferation response to 70% PHx^6^. The cellular proliferation process is extraordinarily complex process that involved variety of signaling pathways, cytokines, transcription factors and chromatin associated proteins^7^. Among these factors, the role of chromatin proteins in the regulation of cell proliferation remains poorly understood.

High-mobility group box 2 (HMGB2) is a chromatin protein that belongs to the HMG protein family and is involved in gene transcription, recombination, and repair processes^8^. HMGB2 is involved in the fine-tuning of gene transcription^9^, and can bend DNA by loosening wrapped DNA and enhance the accessibility of transcription factors and direct protein–protein interactions with specific transcription factors^10^. HMGB2 is highly expressed in all tissues during embryogenesis, but is preserved in only a few tissues in adults, such as testis, ovary and lymphoid tissue^11,12^. HMGB2 is overexpressed in highly proliferative tissues such as testis, ovary, various cancers, and downregulated in senescent cells and aging tissues^13–17^. However, the role of HMGB2 in liver regeneration is largely unknown.

In the present study, we first investigated the functional role of HMGB2 in wild-type (WT) and HMGB2-knock-out (KO) mouse using a 70% PHx model. Immunohistochemistry studies demonstrated the spatiotemporal expression of HMGB2 during liver regeneration, as well as the role of HMGB2 in hepatocyte proliferation using various cell cycle specific markers. Compensatory hypertrophy of hepatocytes was detected by detailed analysis of immunohistochemistry and liver mass measurement. Finally, the in vivo experimental findings were confirmed by in vitro analysis of HMGB2-KO and knock-down mesenchymal stem cells (MSC).

## Results

### Expression of HMGB2 in WT mouse liver

**Figure 1:**
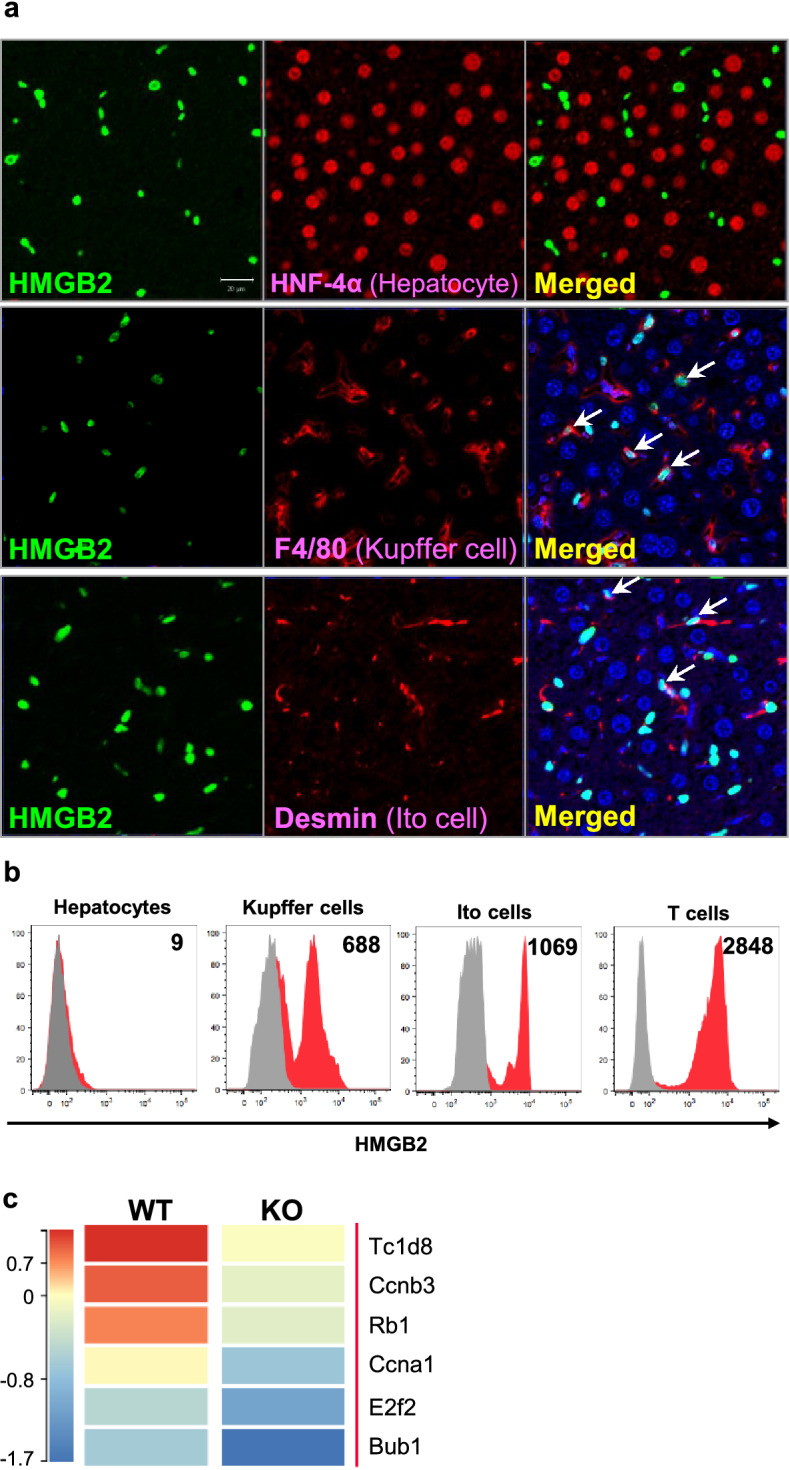
Expression of HMGB2 in mouse liver. (**a**) Double immunofluorescence for HMGB2 (green) and HNF-4α (red staining in upper panel), F4/80 (red staining in middle panel) and desmin (red staining in lower panel), DAPI (blue) in WT mouse liver. Scale bar 20 μm. (**b**) The expressions of HMGB2 and HNF-4α on hepatocytes (CD45-HNF4α + cells) or non-parenchymal liver cells (CD45 + CD3 + T cells, CD45 + , F4/80 + Kupffer cells, CD45-desmin + Ito cells) obtained from WT mice were analyzed by flow cytometry. Gray color indicates unstained cells, whereas, red color indicates double positive cells of HMGB2 and HNF-4α, F4/80, Desmin, CD3. Data are presented by a histogram, and numbers represent mean fluorescence intensity (MFI). (**c**) Heatmap of intact WT and HMGB2-KO mouse livers. Microarray results were analyzed in Transcriptome Analysis Console software (Version 4.0.2.15, Thermo Fisher Scientific) https://www.thermofisher.com/jp/en/home/global/forms/life-science/download-tac-software.html.

First, we examined the presence of HMGB2 in mouse liver. We performed double immunofluorescence using HNF-4α, F4/80 and desmin, which are markers for hepatocytes, macrophages and hepatic stellate cells (Fig. 1a). In normal liver, HMGB2 was co-localized with F4/80 and desmin, but not with HNF-4α. We also examined the expression of HMGB2 in livers at the single-cell level by Flow cytometry. Non-parenchymal cells, such as Kupffer cells, Ito cells and T-cells were expressed HMGB2 comparing to hepatocytes (Fig. 1b). Red colors in histogram indicate the cells that co-expressing HMGB2 and HNF-4a (hepatocyte), F4/80 (Kupffer cells), Desmin (Ito cells) and CD3 (T cells). These results indicate that HMGB2 is expressed in non-parenchymal cells, but not in parenchymal tissues. Moreover, microarray analysis was used to screen for genes that are related to HMGB2 expression (Fig. 1c). The results showed that cell proliferation and cell cycle related genes were downregulated in HMGB2-KO liver compared to WT littermates. Decreased gene expression was observed for *Tc1d8, cyclin B3, Rb1, cyclin A1, E2f2* and *Bub1*, which are crucial regulators of cell cycle progression, especially in the G1/S and G2/M phases.

### Spatiotemporal expression of HMGB2 in hepatocytes during liver regeneration

**Figure 2:**
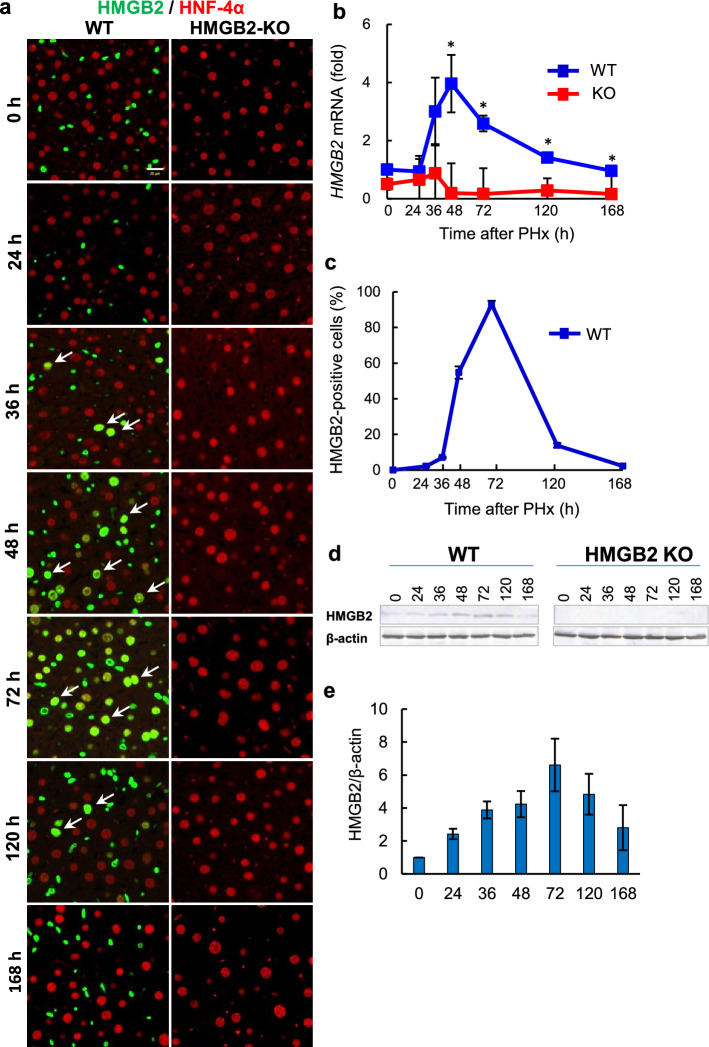
Expression of HMGB2 in mouse liver during regeneration. (**a**) Double immunofluorescence for HMGB2 (green) and HNF-4α (red) in WT and HMGB2-KO mouse livers after PHx. Arrows indicate HMGB2-positive hepatocytes. Scale bar 20 μm. (**b**) qPCR analysis of HMGB2 mRNA during liver regeneration. (**c**) The number of HMGB2-positive hepatocytes was counted at each time-point after PHx. Data represent the mean ± SEM from 5–6 mice per group. (**d**) Western blotting of HMGB2 in WT and HMGB2-KO mouse livers after PHx. (**e**) Densitometry analysis of HMGB2 expression. Data represent the mean ± SEM from 3 mice per group. Asterisks indicate statistically significant differences (*p < 0.05).

We hypothesized that HMGB2 is an important regulator of liver regeneration. Therefore, 70% PHx was performed to induce liver regeneration, and remnant liver tissues were sampled at various time-points. Double immunofluorescence of HMGB2 and HNF-4α was performed to examine the expression of HMGB2 during liver regeneration (Fig. 2a). Interestingly, HMGB2-positive hepatocytes appeared at 36 h, and reached in plateau at 72 h, and disappeared at 168 h after PHx in WT livers. There were no HMGB2-positive cells in HMGB2-KO livers, demonstrating the complete depletion of the target gene. Quantitative analysis of *HMGB2* mRNA (Fig. 2b) and HMGB2-positive cell numbers (Fig. 2c) was used to reveal the spatiotemporal expression of HMGB2 during liver regeneration. Western blotting also confirmed the expression of HMGB2 in normal liver (0 h). The expression of HMGB2 was increased during active cell proliferation and decreased at 120 h and 168 h after PHx (Fig. 2d,e). These results demonstrate that HMGB2 is essential for liver regeneration.

### HMGB2 is important for hepatocyte cell proliferation

**Figure 3:**
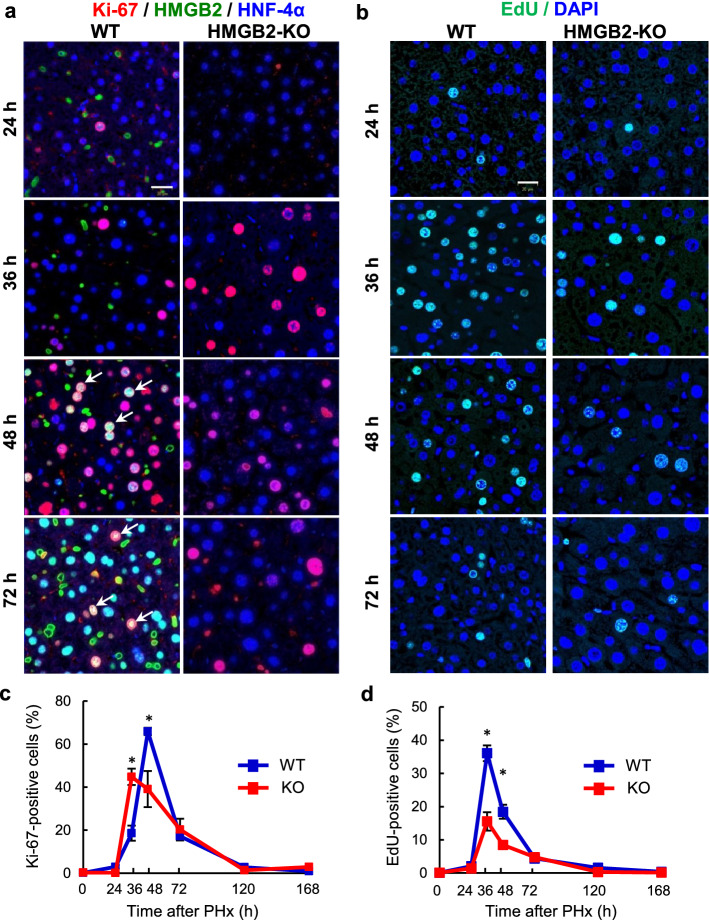
Expression of Ki-67 and EdU during liver regeneration. (**a**) Immunofluorescence for Ki-67 (red), HMGB2 (green) and HNF-4α (blue) in WT and HMGB2-KO mouse livers after PHx. Arrows indicate HMGB2- and Ki-67-positive hepatocytes. (**b**) EdU (green) and DAPI (blue) staining in WT and HMGB2-KO mouse livers after PHx. Scale bar 20 μm. Counting results of Ki-67-positive cells (**c**) and EdU-positive cells (**d**) in WT and HMGB2-KO mouse livers. Data represent the mean ± SEM from 5–6 mice per group. Asterisks indicate statistically significant differences (*p < 0.05).

We further analyzed the role of HMGB2 in hepatocyte cell proliferation using specific cell cycle markers. Co-localization of HNF-4α and Ki-67, which is a marker for dividing cells and is absent in the G_0_ state, was examined (Fig. 3a). In WT livers, the number of Ki-67-positive cells peaked at 48 h, whereas the peak was observed at 36 h in HMGB2-KO livers. Quantitative analysis revealed that the number of proliferating cells was significantly higher in WT livers at 48 h than in HMGB2-KO mice (Fig. 3c). EdU-staining was performed to examine cells in S-phase and the results revealed higher proliferative activity in WT livers than in HMGB2-KO littermates (Fig. 3b, d).

**Figure 4:**
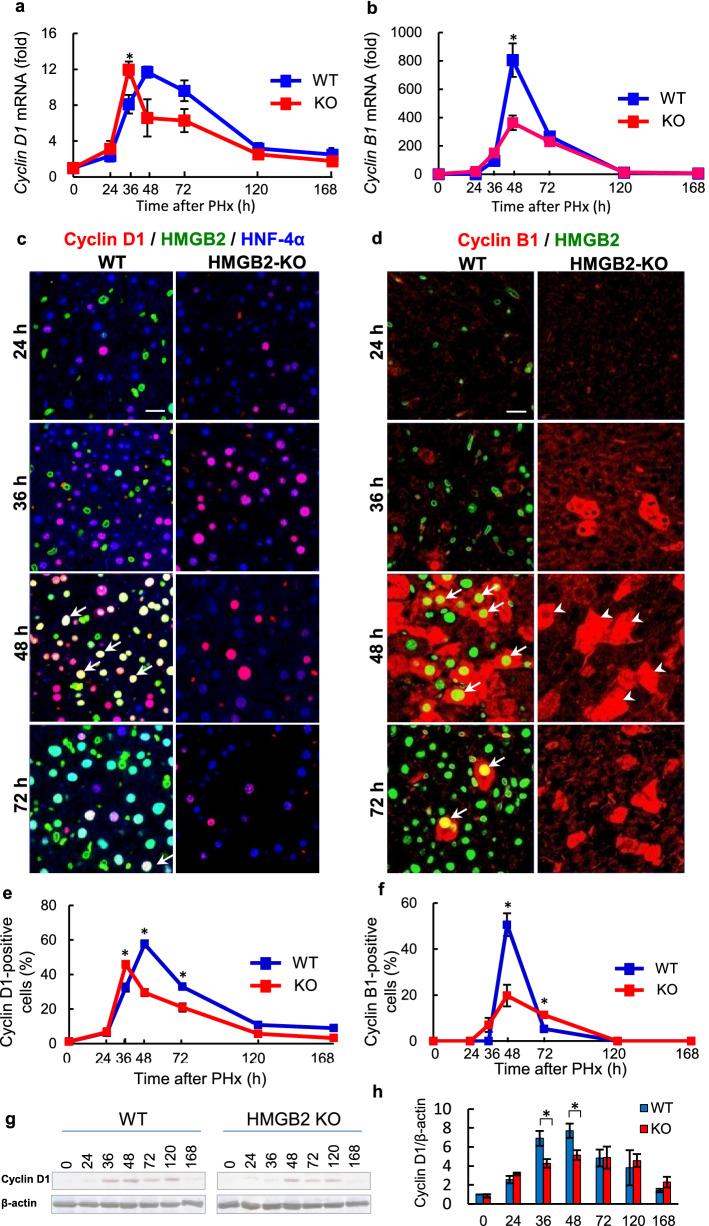
Co-localization of HMGB2 and cyclin D1, cyclin B1 in mouse liver during regeneration. qPCR analysis of *cyclin D1* (**a**) and *cyclin B1* (**b**) mRNA in WT and HMGB2-KO mouse livers. (**c**) Immunofluorescence for cyclin D1 (red), HMGB2 (green) and HNF-4α (blue) in WT and HMGB2-KO mouse livers after PHx. (**d**) Double immunofluorescence for cyclin B1 (red) and HMGB2 (green) in WT and HMGB2-KO mouse livers. Arrows indicate HMGB2- and cyclin D1- or cyclin B1-positive hepatocytes. Arrowheads indicate cyclin B1-positive hepatocytes in HMGB2-KO mouse liver at 48 h. Scale bar 20 μm. The number of cyclin D1- (**e**) and cyclin B1-positive (**f**) hepatocytes was counted at each time-point after PHx. Data represent the mean ± SEM from 5–6 mice per group. (**g**) Western blotting of cyclin D1 in WT and HMGB2-KO mouse livers after PHx. (**h**) Densitometry analysis of cyclin D1 expression. Data represent the mean ± SEM from 3 mice per group. Asterisks indicate statistically significant differences (*p < 0.05).

In general, the G1/S and G2/M phases are crucial for normal cell cycle progression. Therefore, we examined cyclin D1 and cyclin B1, which are specific markers of the G1/S and G2/M phases, respectively. qPCR results revealed significantly decreased expression of *cyclin D1* and *cyclin B1* mRNA in HMGB2-KO liver (Fig. 4a, b). Immunofluorescence results revealed that the number of cyclin D1-positive hepatocytes were significantly higher in WT mice, especially at 48 h after PHx (Fig. 4c, e). Western blotting also confirmed the higher expression of cyclin D1 in WT mouse livers comparing to HMGB2-KO mouse livers (Fig. 4g, h). Next, we analyzed the co-localization of HMGB2 and cyclin B1 in hepatocytes during liver regeneration (Fig. 4d). Surprisingly, the majority of cyclin B1-positive cells were co-stained with HMGB2 in WT liver at 48 h after PHx. The number of cyclin B1-positive cells was significantly fewer in HMGB2-KO livers (Fig. 4f). To confirm these findings, we evaluated the expression of PCNA and Cyclin A2 additional markers of G1/S and G2/M-phase, respectively (Supplementary Fig. S1a–d). PCNA and Cyclin A2 staining resembled observations with cyclin D1 and cyclin B1 (Fig. 4). Western blotting also confirmed the significantly higher expression of PCNA and cyclin A2 in WT mouse livers (Supplementary Fig. S1e–g). pH3S10, a marker of M-phase was demonstrated by double immunofluorescence, and pH3S10-positive cells were significantly higher in WT mouse during active cell proliferation (Supplementary Fig. S2). Altogether, these results suggest that HMGB2 is important for hepatocyte cell proliferation and depletion of HMGB2 leads to delayed cell proliferation.

### Compensatory hypertrophy of hepatocytes in HMGB2-KO mice during liver regeneration

**Figure 5:**
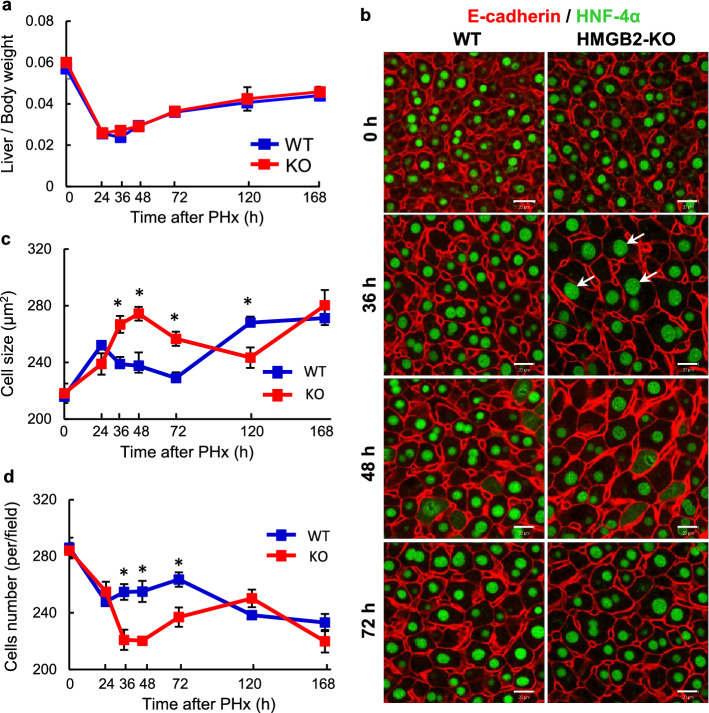
Compensatory hypertrophy during liver regeneration. (**a**) Ratio of mouse liver weight / body weight in WT and HMGB2-KO mouse livers during regeneration. (**b**) Double immunofluorescence for E-Cadherin (red) and HNF-4α (green) in WT and HMGB2-KO mouse livers after PHx. Arrows indicate hypertrophic hepatocytes. Scale bars 20 μm. Dynamic changes of hepatocyte size (**c**) and number (**d**) in WT and HMGB2-KO mouse livers during regeneration. Data represent the mean ± SEM from 5–6 mice per group. Asterisks indicate statistically significant differences (*p < 0.05).

The increase in liver mass during liver regeneration is closely related to cell proliferation^3^. Although a significant delay in cell proliferation was found in HMGB2-KO liver, the observed liver mass was similar to WT mice (Fig. 5a). To investigate this unexpected finding, we performed immunofluorescence for E-cadherin and HNF-4α to examine hepatocyte cell size. Significantly enlarged hepatocytes were found at 36–72 h after PHx in HMGB2-KO mouse liver, with cell size subsequently becoming similar to WT-littermates at 168 h after PHx (Fig. 5b, c). At 36 h after PHx, hepatocyte nuclei size was 50.1 ± 2.5 μm^2^ in WT and 79.2 ± 2.9 μm^2^ in HMGB2-KO, indicating that significantly larger (*p* < 0.0001) nuclei were observed in HMGB2-KO hepatocytes. In contrast, the number of hepatocytes exhibited an opposite trend to cell size (Fig. 5d). Collectively, these results indicate the occurrence of compensatory hepatocyte hypertrophy in HMGB2-KO mice during liver regeneration.

### The importance of HMGB2 in cell proliferation

**Figure 6:**
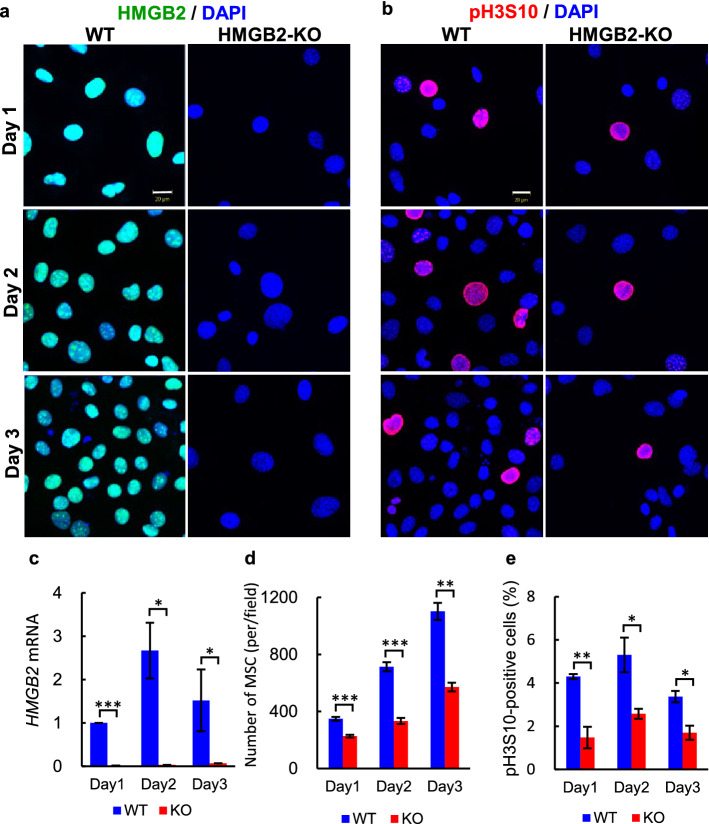
Cell proliferation activity in MSC cells. Immunofluorescence for HMGB2 (**a**) and pH3S10 (**b**) in WT and HMGB2-KO MSCs at 1–3 days post-plating. Nuclei were counterstained with DAPI (blue). Scale bar 20 μm. (**c**) qPCR analysis of *HMGB2* mRNA in WT and HMGB2-KO MSCs. (**d**) Number of MSCs per field were counted in WT and HMGB2-KO cells. (**e**) The percentage of pH3S10-positive cells in WT and HMGB2-KO MSCs. Data represent the mean ± SEM from 4 independent experiments. Asterisks indicate statistically significant differences (*p < 0.05, **p < 0.01, ***p < 0.001).

To verify the in vivo findings described above, we examined the role of HMGB2 in cell proliferation using in vitro experiments. The same number of WT and HMGB2-KO MSCs were seeded and cell proliferation was examined at 1–3 days thereafter. The nuclei of WT-MSCs were mostly positive for HMGB2, whereas HMGB2 was completely absent in HMGB2-KO MSCs, as confirmed by immunocytochemistry and qPCR (Fig. 6a, c). Cellular counting revealed that cell proliferation in HMGB2-KO MSCs was significantly decreased (Fig. 6d). Moreover, pH3S10-positive cells were significantly decreased in cultured HMGB2-KO MSCs (Fig. 6b, e).

**Figure 7:**
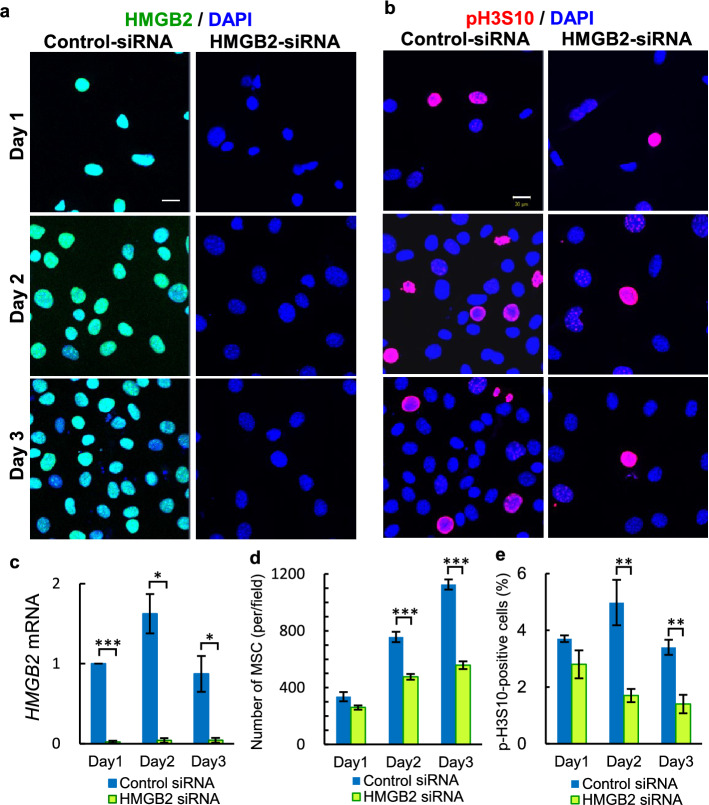
The effect of HMGB2 knockdown on cell proliferation of MSC cells. Immunofluorescence for HMGB2 (**a**) and pH3S10 in (**b**) in control siRNA and HMGB2 siRNA transfected MSCs at 1–3 days post-transfection. Nuclei were counterstained with DAPI (blue). Scale bar 20 μm. (**c**) qPCR analysis of *HMGB2* mRNA in control and HMGB2-knockdown cells. Number of MSCs (**d**) and pH3S10-positive cells (**e**) were counted in control siRNA and HMGB2 knockdown cells. Data represent the mean ± SEM from 4 independent experiments. Asterisks indicate statistically significant differences (*p < 0.05, **p < 0.01, ***p < 0.001).

The role of HMGB2 in cell proliferation was further demonstrated by knockdown experiments. Control- and HMGB2-siRNA were transfected into WT-MSCs, and cell proliferation was analyzed at 1–3 days thereafter. Immunocytochemistry and qPCR experiments confirmed that efficient HMGB2-knockdown occurred in MSCs (Fig. 7a, c). In comparison to control-siRNA transfected cells, the total number of cells was twofold fewer following HMGB2-knockdown cells at day 3 (Fig. 7d). Similarly, pH3S10-positive cells were significantly decreased at day 2 and 3 (Fig. 7b, e). Taken together, the in vivo and in vitro experimental results suggest that HMGB2 is essential for cell proliferation.

## Discussion

This is the first study to investigate the functional role of HMGB2 in mouse liver regeneration. The main findings in this study are the spatiotemporal expression of HMGB2 in proliferating hepatocytes in WT mice, and that the depletion of HMGB2 induced delayed cell proliferation. Moreover, compensatory hepatocyte hypertrophy occurred in HMGB2-KO mouse livers.

The present study revealed that the expression of HMGB2 was limited in non-parenchymal cells, such as Kupffer and stellate cells, but not in hepatocytes under normal conditions. Indeed, hepatocytes are the major parenchymal cells in the liver and play a crucial role in liver metabolism and protein synthesis^18^. Our results indicate that depletion of HMGB2 does not affect major liver function; however, microarray results suggested that the expression of genes related to cell proliferation and the cell cycle were altered. Decreased expression of Rb1, E2f., and cyclin A, cyclin B was found in HMGB2-KO mouse liver, which are pivotal transcription factors in the G1 and G2/M checkpoints, respectively^2^. These findings indicate that cell cycle progression may be altered in the absence of HMGB2. Therefore, we used the PHx mouse model to study liver regeneration and cell cycle dynamics^19,20^.

In WT mouse livers, the spatiotemporal expression of HMGB2 was strongly correlated with active cell proliferation, especially at 36–72 h after PHx. Our results also confirmed that hepatocytes are the first dividing cells, followed by proliferation of cholangiocytes and endothelial cells^21,22^. The expression of Ki-67 is a reliable marker of dividing cells, and it is not expressed in G_0_ phase^23^. Our findings revealed that the number of Ki-67-positive cells was significantly fewer in HMGB2-KO liver, and suggested that depletion of HMGB2 induced an alteration in cell cycle progression. Interestingly, EdU-positive cells were higher than Ki-67-positive cells at 36 h after PHx. These findings could be explained that Ki-67 protein expression is gradually increased from 36 h, and peaked at 48 h, then gradually decreased in later time-points. However, EdU-positive cells corresponds with S-phase of the cell cycle that peaked at 36 h after PHx. Cell cycle checkpoints are a major control mechanism of proper cell cycle progression^2,24^. Although there are many checkpoints, the G1 and G2/M checkpoints play pivotal roles in cell cycle progression, and are strictly regulated by cyclin dependent kinases and their regulatory cyclin subunits^25^. In our study, detailed analyses of cyclin D1, PCNA, EdU and cyclin B1 enabled the analysis of progression through the G1/S and G2 phases, respectively. *Cyclin D1* and *cyclin B1* mRNAs were significantly lower in HMGB2-KO mice during liver regeneration. The qPCR results were confirmed by immunofluorescence, showing that the number of cyclin D1 and cyclin B1-positive cells were significantly fewer in HMGB2-KO liver, indicating that cell cycle progression was delayed in HMGB2 depleted mouse livers after PHx. The expression of pH3S10 represent M-phase of the cell cycle, and number of the pH3S10-positive cells were significantly fewer in HMGB2-KO liver. In addition, partial hepatectomy triggers the activation of hepatic stellate cells and Kupffer cells which are important to promote exit from G_0_ state of hepatocytes. Therefore, we are hypothesizing that the absence of HMGB2 in hepatic stellate cells and Kupffer cells may affect in delayed cell proliferation in HMGB2 KO mice after partial hepatectomy^26^.

The number of Ki-67, cyclin D1, PCNA-positive cells was higher at 36 h after PHx, indicating that cell proliferation seems to be initiated early in HMGB2-KO mice. However, the number of proliferating cells were significantly higher in WT livers 48 h after PHx, which is the peak period for cell proliferation^27,28^. At 36 h after PHx, the expression of PCNA and Cyclin D1 were higher in HMGB2 KO mouse liver by immunofluorescence, whereas western blotting detected higher expression in WT mouse livers. This discrepancy may be explained by that HNF-4a-positive hepatocytes were counted in immunofluorescence, while total tissue lysate is analyzed by western blotting. In vitro experiments demonstrated that cell proliferation was significantly decreased in both HMGB2-KO and knockdown cells. Our results were consistent with those of Bagherpoor et al^29^, namely that HMGB2-knockdown downregulates cell proliferation and the efficiency of differentiation in human embryonic stem cells. Detailed cell cycle analysis suggested that the cell cycle is delayed, but not arrested in HMGB2-KO mouse liver. Our results revealed that the cell proliferation was peaked at 48 h, whereas HMGB2 expression was peaked at 72 h after PHx. Taken together, these results suggest that HMGB2 plays an essential role in hepatocyte proliferation.

In general, hypertrophy is a compensatory response to partial loss of a tissue or organ. In various organs, such as the kidney, compensatory hypertrophy is observed after partial and radical unilateral nephrectomy^30^. Moreover, the grade of hypertrophy is strongly correlated with the amount of excised parenchymal volume of the kidney^31^. In addition, a similar finding was observed in skeletal muscle, namely that the rapid enlargement of muscle occurs in response to removal of synergistic muscles^32^. Unlike other organs, the liver has extraordinary potential to regenerate by means of both hypertrophy and cell proliferation^5,33^. Although cell proliferation was altered in HMGB2-KO mice, a greater extent of liver hypertrophy was observed at 36–72 h after PHx compared to WT littermates. These results suggest that compensatory hypertrophy of hepatocytes occurs during liver regeneration in HMGB2 depleted mice. Similar findings of compensatory hypertrophy and altered cell proliferation were also observed in the Stat3, Skp2 and Separase-deficient mouse liver^34–36^. Thus, it seems that there is no single protein or pathway that completely stops liver regeneration^33^.

In the present study, the hypertrophic hepatocytes contained significantly enlarged nuclei in HMGB2-KO livers compared to WT-littermates. This finding could be explained by the nucleoskeletal theory, where the amount of DNA influences nuclear volume as well as affecting cell size^37,38^. Nuclear size is proportional to the amount of DNA, and it has a pivotal role in gene expression and transcription^39^. We hypothesized that the enlarged nuclei in hepatocytes may appear as a result of delayed cell cycle progression in HMGB2-KO liver. Our results suggest that enlarged hepatocyte nuclei is closely associated with the timing of most active cell proliferation. Miyaoka et al.^5^ also reported that enlarged nuclei in hypertrophic hepatocytes was observed during liver regeneration after 70% PHx in mice.

In conclusion, the present study demonstrated that HMGB2 depletion causes delayed cell proliferation as well as compensatory hypertrophy of hepatocytes during liver regeneration, suggesting that HMGB2 is essential for proper cell proliferation.

## Materials and methods

### Chemicals and biochemicals

Paraformaldehyde (PFA) was purchased from Merck (Darmstadt, Germany). Trizma base, bovine serum albumin (BSA), 3-aminopropyl-triethoxysilane, Triton X-100, Tyramine hydrochloride and Brij L23 were purchased from Sigma Chemical Co. (St Louis, MO, USA). Clariom S microarray chip for mouse, click-iT Cell Reaction Buffer Kit, FITC- and rhodamine-succinimidyl esters and Lipofectamine RNA iMAX were purchased from Invitrogen (California, USA). 5-Ethynyl-2'-deoxyuridine (EdU) was purchased from TCI chemicals (Tokyo, Japan). All other reagents used in this study were purchased from Fujifilm Wako Pure Chemicals (Osaka, Japan).

### Animals and tissue preparation

Male C57BL/6 WT and HMGB2-KO mice (8 weeks old) were used in the present study. The derivation of genomic HMGB2-KO mouse has been described previously^14^. Mice were fed normal chow and allowed to drink water ad libitum. The experimental protocol was approved by the Animal Ethics Review Committee of the University of Miyazaki (2018-510-7) and all experiments were performed in accordance with institutional guidelines of the Animal Experiment Committee. The ARRIVE Essential 10 guideline was used to formulate study design, sample preparation, result observation and data analysis. Mice were anesthetized by inhalation of isoflurane and 70% PHx was performed using a previously described technique^3,40^. EdU was injected 2 h prior to sampling. After PHx, mice were sacrificed at 0, 24, 36, 48, 72, 120 and 168 h, and tissues were sampled as described below. Liver tissue was cut into several small pieces and some were snap frozen and kept at − 80 °C until use in microarray, quantitative-polymerase chain reaction (qPCR) and western blotting analysis. The remaining pieces of liver tissue were fixed overnight in 4% PFA in phosphate-buffered saline (PBS) at room temperature and subsequently embedded in paraffin using standard methods. Five to six mice were used in each experimental group.

### Immunohistochemistry

Paraffin-embedded tissues were cut into 3-µm-thick sections and placed onto silane-coated slide glasses. The sections were deparaffinized with toluene and rehydrated using a graded ethanol series, then autoclaved at 120 °C for 15 min in 10 mM citrate buffer (pH 6.0). After inhibition of endogenous peroxidase activity with 3% H_2_O_2_ in methanol for 30 min, the sections were pre-incubated with 500 µg/ml normal goat IgG and 1% BSA in PBS for 1 h to block non-specific binding of antibodies. The sections were then reacted with the following primary antibodies for 16–17 h: anti-HMGB2 (Abcam, ab124670), anti-HNF-4α (Abcam, ab181604), anti-F4/80 (Abcam, ab6640), anti-Desmin (Dako, IS606), anti-PCNA (Dako, M0879), anti-Ki-67 (Dako, M7248), anti-Cyclin D1 (Cell signaling, #2978), anti-Cyclin B1 (Abcam, ab181593), anti-phosphorylated H3S10 (Cell Signaling, #9708) and anti-E-Cadherin (Novus Bio, NBP2-19051). After washing with 0.075% Brij L23 in PBS, the sections were reacted with HRP-goat anti-mouse IgG or HRP-goat anti-rabbit IgG for 1 h. After washing in 0.075% Brij L23 in PBS, the HRP-sites were visualized with FITC-conjugated tyramide, then microwaved at 95ºC for 15 min in 10 mM citrate buffer (pH 6.0). Then, next primary antibody was reacted for overnight and repeated its detection with rhodamine conjugated tyramide and then counterstained with DAPI^12,41^. In some experiments, HNF-4α was visualized by Alexa-633 conjugated goat anti-rabbit IgG. As a negative control, normal mouse or rabbit IgG was used at the same concentration instead of the primary antibody in each experiment. EdU was detected using the click-iT Cell Reaction Buffer Kit, according to manufacturer’s instruction. EdU-positive hepatocytes were referred as cells with large round nuclei in DAPI staining. Microphotographs were obtained using a laser scanning microscope (Zeiss LSM700) and a light microscope (Olympus BX53) with a fluorescence camera (DP-74, Olympus, Japan).

### Flow cytometry

Normal mouse livers (n = 3) were perfused with PBS and then digested with 400 U/ml collagenase type III (Worthington Biochemical) at 37 °C for 30 min. The digested cells were passed through a 100-μm cell strainer by forcing and washed with PBS. The cell suspension was centrifuged at 50 g for 3 min for hepatocyte enrichment. The pellet and supernatant were used as the hepatocyte enrichment fraction and non-parenchymal liver cells, respectively. Cells were stained with fluorescein-conjugated mAbs (1:200) to mouse CD3ε (Brilliant Violet 421, 146-2C11; Biolegend), mouse CD45 (APC/Cyanine7, 30-F11; Biolegend), mouse F4/80 (APC, BM8; Biolegend). For intracellular staining, cells were fixed and permeabilized with Foxp3/Transcription factor staining buffer set (Invitrogen) according to the manufacturer’s instructions. Subsequently, the cells were stained with mAbs (1:200) to desmin, HNF-4α and HMGB2, followed by anti-rabbit IgG Alexa Fluor 488, anti-rabbit IgG Alexa Fluor 633, or anti-mouse IgG Alexa Fluor 633 secondary antibody. Fluorescence staining was analyzed with a FACSVerse flow cytometer (BD Biosciences) and FlowJo software (Tree star).

### qPCR analysis

Total RNA was extracted from snap frozen liver tissues using Isogen II (Nippon Gene, Tokyo, Japan), as reported previously^42^. RNA was reverse transcribed to cDNA using Moloney murine leukemia virus reverse transcriptase (Invitrogen, Carlsbad, CA, USA). Transcript expression levels were analyzed by an ABI StepOne plus Real-Time PCR System (Applied Biosystems, Waltham, MA, USA) using Fast SYBR Green (Applied Biosystems). β-actin was used for normalization and relative gene expression was calculated using the 2 − ΔΔct method. The primer pairs were listed below. HMGB2 F-5′-TCCTGGTAGGCCAACAGGCT-3′ and R-5′-AGCTAATGTTGAGCTGCACTTG-3′, Cyclin B1 F-5′-AGAGGTGGAACTTGCTGAGCCT-3′, R-5′-GCACATCCAGATGTTTCCATCGG-3′, Cyclin D1 F-5′-GCGTACCCTGACACCAATCTC-3′, R-5′-CTCCTCTTCGCACTTCTGCTC-3′, and β-actin F-5′- TCCTCCCTGGAGAAGAGCTAC -3′, R-5′- TCCTGCTTGCTGATCCACAT -3′.

### Microarray

RNA was isolated from WT and HMGB2 KO mouse livers (n = 3 each), and cRNA was prepared as described previously^43,44^. Fifteen micrograms of fragmented cRNA from each sample was hybridized to a pre-equilibrated Clariom S mouse microarray chip, which was then washed, stained, and scanned in an HP ChipScanner (Affymetrix Inc., Santa Clara, USA). Data normalization was performed using GeneSpring (Agilent Technologies, Santa Clara, CA, USA). All entities (22,206 genes) were filtered based on significant changes in gene expression between WT and HMGB2 KO mouse livers. Data were analyzed in Transcriptome Analysis Console software (Version 4.0.2.15, Thermo Fisher Scientific).

### Cell culture

Bone marrow-derived MSCs were prepared from the tibias and femurs of 6- to 8-week-old C57BL/6J WT and HMGB KO mice, as described previously^24,45^. Cells were cultured with 10% FBS/DMEM.WT-MSCs were divided into two groups, and transfected with HMGB2-small interfering RNA (siRNA) (Integrated DNA Technologies, MMC.RNAI.N008252.3_2 nm, Coralville, IA, USA) or control siRNA (Integrated DNA Technologies, DS Scrambled-Neg universal negative control duplex) using Lipofectamine RNA iMAX, as described previously^46^. Successfully transfected cells were prepared into a cell suspension using α-MEM with L-Glutamine and Phenol Red with 10% FBS, 50 µg/ml ascorbic acid, and 5 mM β-glycerophosphate. The cells were seeded at a density of 1 × 10^4^ cells per well in 24-well plates, followed by incubation in a constant temperature incubator (5% CO_2_, 37 °C, 95% humidity). The cells were then analyzed by qPCR and immunocytochemistry at 1–3 days.

### Western blot analysis

Liver tissues were homogenized in hot SDS lysis buffer with a glass-teflon homogenizer, as described previously^12,47^. After centrifugation of the homogenate at 15,000 rpm for 30 min at 4 °C, the supernatant was collected and stored at − 80 °C. The protein concentration in each preparation was determined using a BCA assay kit. Lysate containing 20 µg of protein was separated by 10% SDS-PAGE, and the proteins were electrophoretically transferred onto PVDF membranes. The membranes were blocked with 5% nonfat milk in Tris-buffered saline (TBS; 20 mM Tris buffer [pH 7.6], 150 mM NaCl) for 1 h at RT and then incubated overnight with anti-HMGB2 or anti-HMGB1 antibodies diluted 1:1000 with TBS/0.05% Triton X-100 buffer. As a secondary antibody, HRP-goat anti-rabbit IgG or HRP-goat anti-mouse IgG was diluted with TBS buffer for 1 h, and the membranes were washed 3 times for 10 min each with TBS/0.05% Triton X-100 buffer. Bands were visualized with DAB, Ni, Co, and H_2_O_2_. Densitometric analysis was performed using ImageQuant LAS 4000 (GE Healthcare, Fairfield, CT, USA). β-Actin was used as an internal standard in each lane for normalization of target protein expression.

### Quantitative analysis

The number of hepatocytes and proliferating cells was counted in 10 random high-magnification fields per mouse using ImageJ software (NIH, Bethesda, Maryland, USA) . The size of hepatocyte nuclei stained by HNF-4α was measured in 5 high-magnification fields per mouse using analyze particles tool in ImageJ software.

### Statistical analysis

All data are expressed as mean ± standard error of the mean (SEM). Statistical significance was assessed using the Student’s *t*-test. *P* < 0.05 was considered to be statistically significant. All analyses were performed with JMP (version 15.1.0, SAS Institute Inc., Cary, NC, USA).

## Supplementary Information

Supplementary Information.

## Data Availability

Original micrographs and any other information are available upon request from the corresponding author.

## References

[1] Michalopoulos GK, Bhushan B (2021). Liver regeneration: biological and pathological mechanisms and implications. Nat. Rev. Gastroenterol. Hepatol..

[2] Nevzorova, Y. A. & Trautwein, C. *Liver Regeneration* 153–166, DOI: 10.1016/B978-0-12-420128-6.00011-7 (2015)..

[3] Batmunkh B (2017). Estrogen accelerates cell proliferation through estrogen receptor alpha during rat liver regeneration after partial hepatectomy. Acta Histochem. Cytochem.

[4] Meier M (2016). Liver regeneration is dependent on the extent of hepatectomy. J. Surg. Res..

[5] Miyaoka Y (2012). Hypertrophy and unconventional cell division of hepatocytes underlie liver regeneration. Curr. Biol..

[6] An S, Soe K, Akamatsu M, Hishikawa Y, Koji T (2012). Accelerated proliferation of hepatocytes in rats with iron overload after partial hepatectomy. Histochem. Cell Biol..

[7] Miyaoka Y, Miyajima A (2013). To divide or not to divide: revisiting liver regeneration. Cell Div..

[8] Chen K (2021). HMGB2 orchestrates mitotic clonal expansion by binding to the promoter of C/EBPβ to facilitate adipogenesis. Cell Death Dis..

[9] Ueda T, Yoshida M (2010). HMGB proteins and transcriptional regulation. Biochim. Biophys. Acta..

[10] Bianchi ME, Agresti A (2005). HMG proteins: dynamic players in gene regulation and differentiation. Curr. Opin. Genet. Dev..

[11] Yamaguma Y (2022). Crucial role of high-mobility group box 2 in mouse ovarian follicular development through estrogen receptor beta. Histochem Cell Biol..

[12] Sugita N (2021). Depletion of high-mobility group box 2 causes seminiferous tubule atrophy via aberrant expression of androgen and estrogen receptors in mouse testis. Biol. Reprod..

[13] Taniguchi N (2009). Aging-related loss of the chromatin protein HMGB2 in articular cartilage is linked to reduced cellularity and osteoarthritis. Proc. Natl. Acad. Sci. U S A.

[14] Ronfani L (2001). Reduced fertility and spermatogenesis defects in mice lacking chromosomal protein Hmgb2. Development.

[15] Zirkel A (2018). HMGB2 loss upon senescence entry disrupts genomic organization and induces CTCF clustering across cell types. Mol. Cell..

[16] Fu D (2018). HMGB2 is associated with malignancy and regulates Warburg effect by targeting LDHB and FBP1 in breast cancer. Cell Commun. Signal.

[17] Guerrero A, Gil J (2016). HMGB2 holds the key to the senescence-associated secretory phenotype. J. Cell Biol..

[18] Zhou Z, Xu MJ, Gao B (2016). Hepatocytes: a key cell type for innate immunity. Cell Mol. Immunol..

[19] Pritchard, M. T. & Apte, U. *Liver Regeneration* 15–40 10.1016/B978-0-12-420128-6.00002-6 (2015).

[20] Furchtgott LA, Chow CC, Periwal V (2009). A model of liver regeneration. Biophys. J..

[21] De Rudder M, Dili A, Starkel P, Leclercq IA (2021). Critical role of LSEC in post-hepatectomy liver regeneration and failure. Int. J. Mol. Sci..

[22] Abu Rmilah A (2019). Understanding the marvels behind liver regeneration. Wiley Interdiscip Rev. Dev. Biol..

[23] Uxa S (2021). Ki-67 gene expression. Cell Death Differ..

[24] Engeland K (2018). Cell cycle arrest through indirect transcriptional repression by p53: I have a DREAM. Cell Death Differ..

[25] Wang Z (2021). Regulation of cell cycle progression by growth factor-induced cell signaling. Cells.

[26] Greenbaum LE (2004). Cell cycle regulation and hepatocarcinogenesis. Cancer Biol. Ther..

[27] Minocha S (2017). Segregated hepatocyte proliferation and metabolic states within the regenerating mouse liver. Hepatol. Commun..

[28] Rizvi F (2021). Murine liver repair via transient activation of regenerative pathways in hepatocytes using lipid nanoparticle-complexed nucleoside-modified mRNA. Nat. Commun..

[29] Bagherpoor AJ (2017). Properties of human embryonic stem cells and their differentiated derivatives depend on nonhistone DNA-binding HMGB1 and HMGB2 proteins. Stem Cells Dev..

[30] Rojas-Canales DM, Li JY, Makuei L, Gleadle JM (2019). Compensatory renal hypertrophy following nephrectomy: When and how?. Nephrology (Carlton).

[31] Takagi T (2014). Compensatory hypertrophy after partial and radical nephrectomy in adults. J. Urol..

[32] Armstrong RB, Marum P, Tullson P, Saubert CW (1979). Acute hypertrophic response of skeletal muscle to removal of synergists. J. Appl. Physiol. Respir. Environ. Exerc. Physiol..

[33] Yagi S, Hirata M, Miyachi Y, Uemoto S (2020). Liver regeneration after hepatectomy and partial liver transplantation. Int. J. Mol. Sci..

[34] Haga S (2005). Compensatory recovery of liver mass by Akt-mediated hepatocellular hypertrophy in liver-specific STAT3-deficient mice. J. Hepatol..

[35] Minamishima YA, Nakayama K, Nakayama K (2002). Recovery of liver mass without proliferation of hepatocytes after partial hepatectomy in Skp2-deficient mice. Cancer Res..

[36] Wirth KG (2006). Separase: a universal trigger for sister chromatid disjunction but not chromosome cycle progression. J. Cell Biol..

[37] Wu Y, Pegoraro AF, Weitz DA, Janmey P, Sun SX (2022). The correlation between cell and nucleus size is explained by an eukaryotic cell growth model. PLoS Comput. Biol..

[38] Webster M, Witkin KL, Cohen-Fix O (2009). Sizing up the nucleus: nuclear shape, size and nuclear-envelope assembly. J. Cell Sci..

[39] Mukherjee RN (2020). The perinuclear ER scales nuclear size independently of cell size in early embryos. Dev. Cell.

[40] Anderson R, Higgins G (1931). Experimental pathology of the liver. 1. Restoration of the liver of the white rat following partial surgical removal. Arch. Pathol..

[41] Buchwalow I, Samoilova V, Boecker W, Tiemann M (2018). Multiple immunolabeling with antibodies from the same host species in combination with tyramide signal amplification. Acta Histochem. Cytochem..

[42] Srisowanna N (2019). The effect of estrogen on hepatic fat accumulation during early phase of liver regeneration after partial hepatectomy in rats. Acta Histochem. Cytochem..

[43] Saha HR (2018). Suppression of GPR56 expression by pyrrole-imidazole polyamide represents a novel therapeutic drug for AML with high EVI1 expression. Sci. Rep..

[44] Nakahata S (2014). Loss of NDRG2 expression activates PI3K-AKT signalling via PTEN phosphorylation in ATLL and other cancers. Nat. Commun..

[45] Taniguchi N (2011). Expression patterns and function of chromatin protein HMGB2 during mesenchymal stem cell differentiation. J. Biol. Chem..

[46] Lee D (2018). HMGB2 is a novel adipogenic factor that regulates ectopic fat infiltration in skeletal muscles. Sci. Rep..

[47] Mai NNH (2020). Photodynamic therapy using a novel phosphorus tetraphenylporphyrin induces an anticancer effect via Bax/Bcl-xL-related mitochondrial apoptosis in biliary cancer cells. Acta Histochem. Cytochem..

