# Supplementary material for: Spatiotemporal expression of HMGB2 regulates cell proliferation and hepatocyte size during liver regeneration
Source: Sci Rep. 2022 Jul 13;12:11962. doi: 10.1038/s41598-022-16258-4 (PMC9279446; doi:10.1038/s41598-022-16258-4)
Supplement: Supplementary file 1 — Supplementary Information. [file 41598_2022_16258_MOESM1_ESM.pdf]

Spatiotemporal expression of HMGB2 regulates cell proliferation and hepatocyte size during liver regeneration

Koichi Yano[1,2], Narantsog Choijookhuu[1], Makoto Ikenoue[1,2], Fidya Fidya[1], Tomohiro Fukaya[3], Katsuaki Sato[3], Deokcheol Lee[4],Noboru Taniguchi[5], Etsuo Chosa[4], Atsushi Nanashima[2] and Yoshitaka Hishikawa[1*]

[1]Department of Anatomy, Histochemistry and Cell Biology, Faculty of Medicine, University of Miyazaki, 5200 Kihara, Kiyotake, Miyazaki 889-1692, Japan

[2]Department of Surgery, Faculty of Medicine, University of Miyazaki, Miyazaki 889-1692, Japan

[3]Division of Immunology, Department of Infectious Diseases, Faculty of Medicine, University of Miyazaki, Miyazaki, Japan

[4]Department of Orthopaedic Surgery, Faculty of Medicine, University of Miyazaki, 5200 Kihara, Kiyotake, Miyazaki 889-1692, Japan

[5]Department of Orthopaedic Surgery, Graduate School of Medical and Dental Sciences, Kagoshima University, 8-35-1 Sakuragaoka, Kagoshima 890-8520, Japan

Running title: The role of HMGB2 during liver regeneration

Correspondence:

*Yoshitaka Hishikawa, M.D., Ph.D.,

Department of Anatomy, Histochemistry and Cell Biology, Faculty of Medicine,

University of Miyazaki, 5200 Kihara, Kiyotake, Miyazaki 889-1692, Japan. Tel: +81-985-85-1783. Fax: +81-985-85-9851 E-mail: yhishi@med.miyazaki-u.ac.jp

Supplementary figure S1

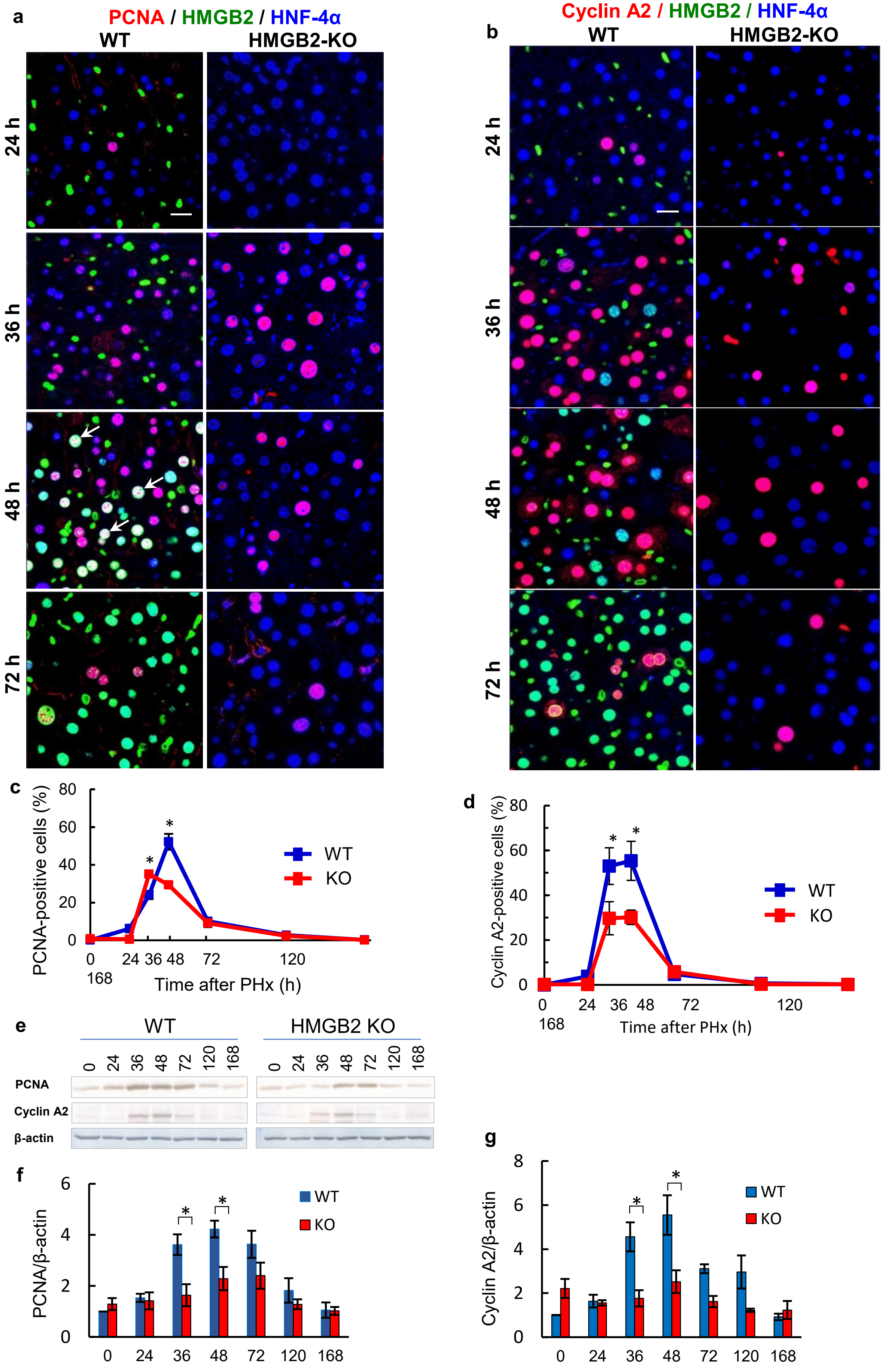

Supplementary figure S2

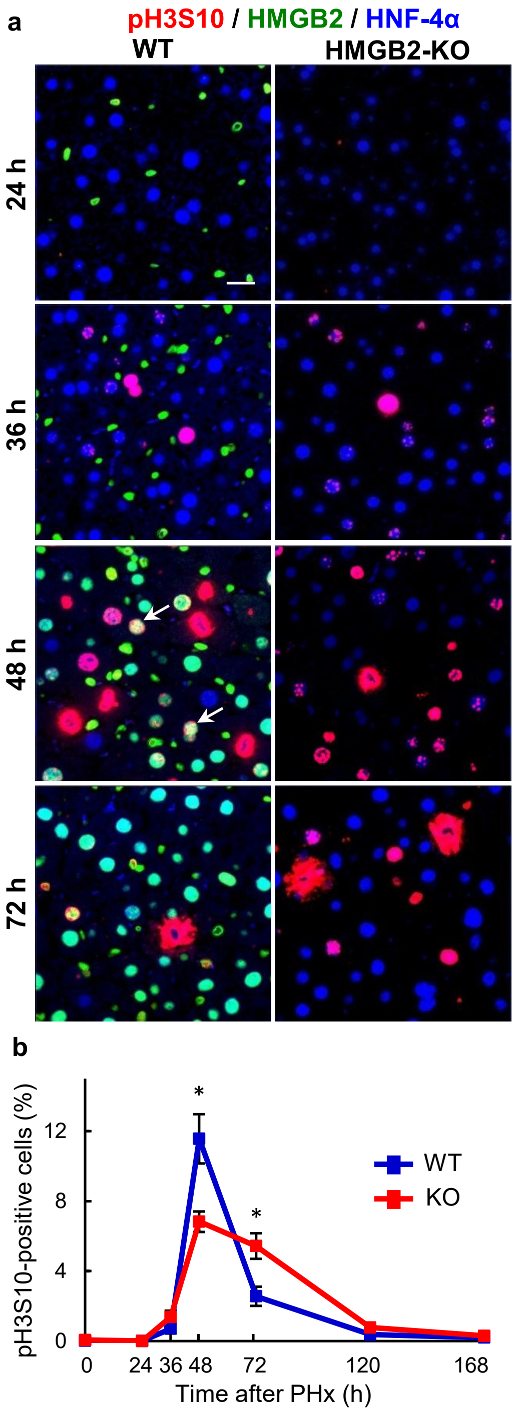

Supplementary figure S3

a

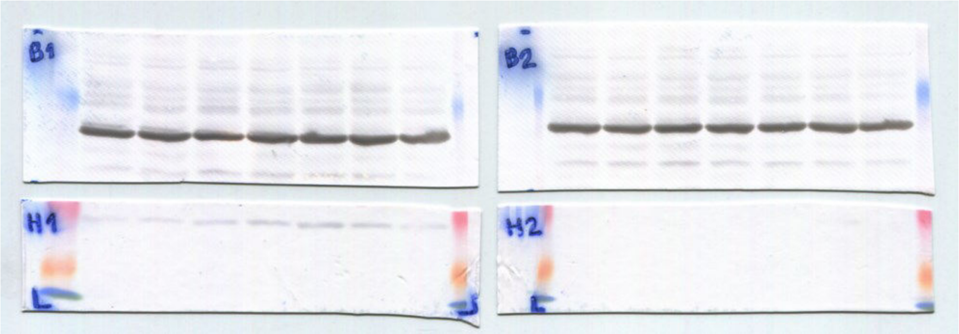

b

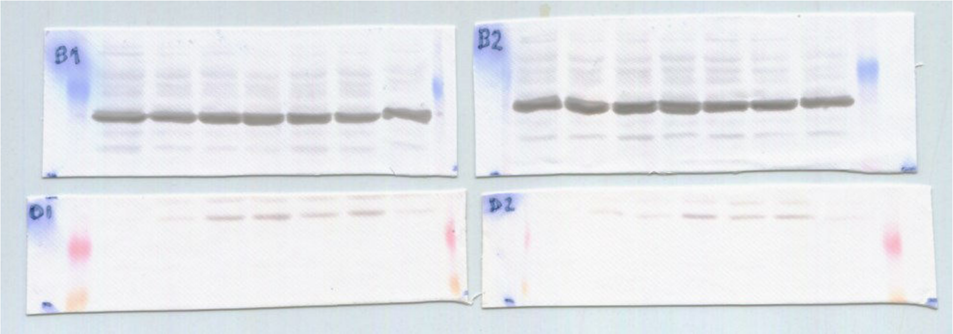

c

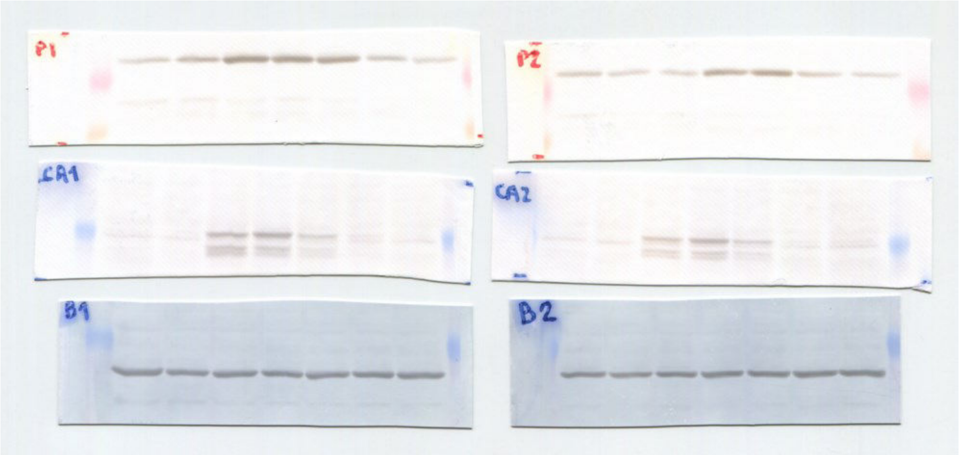

Supplementary figure S1.

PCNA and Cyclin A2 expression in mouse liver during regeneration. (**a**) Immunofluorescence for PCNA (red), HMGB2 (green) and HNF-4α (blue) in WT and HMGB2-KO mouse livers after PHx. (**b**) Immunofluorescence for Cyclin A2 (red), HMGB2 (green) and HNF-4α (blue) in WT and HMGB2-KO mouse livers after PHx. Arrows indicate HMGB2- and PCNA-positive hepatocytes. Scale bar 20 μm. Counting results of PCNA- (**c**) and Cyclin A2-positive (**d**) cells in WT and HMGB2-KO mouse livers after PHx. Data represent the mean ± SEM from 5-6 mice per group. (**e**) Western blotting of PCNA and Cyclin A2 in WT and HMGB2-KO mouse livers after PHx. Densitometry analysis of PCNA (**f**) and Cyclin A2 (**g**). Data represent the mean ± SEM from 3 mice per group. Asterisks indicate statistically significant differences (*$p<0.05$).

Supplementary figure S2.

pH3S10 expression in mouse liver during regeneration. (**a**) Immunofluorescence for pH3S10 (red), HMGB2 (green) and HNF-4α (blue) in WT and HMGB2-KO mouse livers after PHx. Arrows indicate HMGB2- and pH3S10-positive hepatocytes. Scale bar 20 μm. (**b**) Counting results of pH3S10-positive cells in WT and HMGB2-KO mouse livers after PHx. Data represent the mean ± SEM from 5-6 mice per group. Asterisks indicate statistically significant differences (*$p<0.05$).

Supplementary figure S3.

Original images of western blotting that revealed in Fig. 2d, Fig. 4g and Supplementary figure S1e. Representative images of three independent experiments are revealed.
